# Mitochondria in oocyte aging: current understanding

**Published:** 2017-03

**Authors:** D Zhang, D Keilty, ZF Zhang, RC Chian

**Affiliations:** State Key Laboratory of Reproductive Medicine, Nanjing Medical University, Nanjing, P. R. of China; Hangzhou Women’s Hospital, Hangzhou, P. R. of China; Department of Obstetrics and Gynecology, McGill University, Montreal, Canada.

**Keywords:** Oocyte, aging, mitochondria, mtDNA, embryo

## Abstract

The oocyte is the largest cell found in multicellular organisms. Mitochondria, as the energy factories for cells, are found in high numbers in oocytes, as they provide the energy for oocyte maturation, fertilization, and embryo formation via oxidative phosphorylation. Failure of assisted reproduction is mainly attributed to oocyte aging and increased aneuploidy. As the most numerous organelle in the oocyte, the mitochondrion has been confirmed as a crucial player in the process of oocyte aging, which is highly influenced by mitochondrion dysfunction. Every mitochondrion contains one or more mitochondrial DNA (mtDNA) molecule, which, at about 16.5 KD in length, encodes 13 proteins. In this review, we discuss the function of mitochondria and the relationship between mtDNA and oocyte aging. We also discuss technologies that aim to enhance oocyte developmental potential and delay ovarian aging.

## Introduction

As women age, their reproductive capacity decreases (Chappel, 2013), and even in assisted reproduction programs, both the pregnancy and implantation rate of women of advanced reproductive age (ARA) are much lower than that of younger patients. The main reasons for this decrease are decreased oocyte number and quality and falling hormone levels. The oocyte is the largest cell in multicellular organisms, and more mitochondria are found per oocyte than any other cellular organelle. Mitochondria provide energy for transcription and translation during oocyte maturation, fertilization, and embryonic development (May-Panloup et al., 2007). After fertilization, the mitochondria of the sperm are rapidly degraded, and so embryonic mitochondria are derived exclusively from the oocyte. The quality of oocyte mitochondria thus determines the quality of the embryo. Notably, mistakes in chromosome segregation in the oocyte, particularly meiotic nondisjunction, occur more frequently in ARA women (Rabinowitz et al., 2012). The role of mitochondria in oocyte aging has thus long been a popular research topic.

## 1. Mitochondria and mitochondrial DNA

### 1.1. Mitochondria

**Figure 1:**
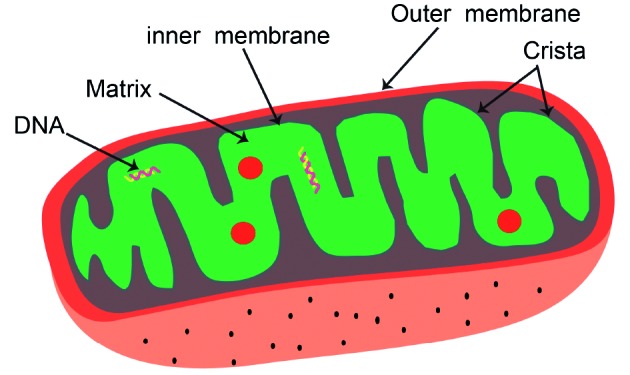
— Mitochondrial structure. A cross-sectional view of a mitochondrial tubule reveals four distinct parts: an intermembrane space between an outer membrane and inner membrane, and a compartment enclosed by the inner membrane called the matrix. Within the matrix, mitochondrial DNA (mtDNA) are attached to the inner membrane.

Mitochondria are double membrane-bound organelles with a highly specialized function and morphology. A cross-sectional view of a mitochondrial tubule reveals four distinct parts: an inter-membrane space between an outer membrane and inner membrane, and a compartment enclosed by the inner membrane called the matrix. Within the matrix, mitochondrial DNA (mtDNA) are attached to the inner membrane (Fig.1). Mitochondrial division is not coupled to cellular division, so mtDNA replicates independently. Mitochondria produce essential energy for diverse cellular functions (Saraste, 1999; Kelly et al., 2004): they are critical for metabolism, signalling, and programmed cell death (Butow et al., 2004; Danial et al., 2004). Oocyte mitochondria generate ATP via oxidative phosphorylation, providing the energy required from fertilization through the blastocyst stage (Van Blerkom et al., 1995; Dumollard et al., 2007).

A number of studies have shown that mitochondrial morphology is complex and plastic. In various cell types, mitochondria are commonly interconnected by reshaping of their elongated tubules (Hoffmann et al.,1973; Bereiter-Hahn et al., 1994; Nunnari et al., 1997). They often alter their shape and size, and travel long distances along cytoskeletal tracks. In response to changing intracellular and extracellular needs, mitochondrial function is optimized by sophisticated mechanisms that regulate different morphologies and distributions. Mitochondrial damage and oxidative stress are the cause of cellular aging in a wide variety of cell types (Liochev, 2013).

### 1.2. Mitochondrial DNA

**Figure 2:**
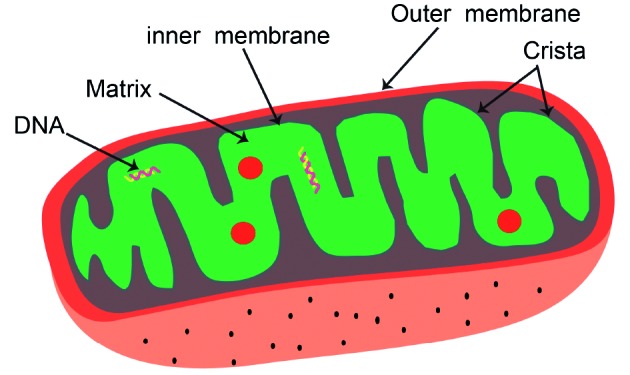
— Mitochondrial genome. The mitochondrial genome encodes 13 of the subunits of the electron transfer chain. These include 7 subunits of Complex I, 1 subunit of Complex III, 3 subunits of Complex IV and 2 subunits of ATP synthase. The mitochondrial genome encodes the core complexes for cellular respiration: 2 subunits of ATP synthase, 3 subunits of Complex IV (cytochrome c oxidase), 1 subunit of Complex III (cytochrome b), and seven subunits of complex I.

Every mitochondrion contains 1 to 15 mtDNA molecules. mtDNA copy number per oocyte has been shown to be highly associated with the probability of developing a healthy oosperm (May-Panloup et al., 2007; Wai et al., 2010). The mammalian mitochondrial genome is a circular double-stranded DNA of 16,569 base pairs (bp) encoding 37 genes, divided into gene-encoding and non-encoding areas. Each gene has its own promoter and contains no introns. The mitochondrial genome encodes the core complexes for cellular respiration: (Stojkovic et al., 2001; Kogo et al., 2011) two subunits of ATP synthase, cytochrome c oxidase, cytochrome b, and seven subunits of complex I (also known as NADH dehydrogenase) (Fig.2). The cell’s nuclear genome encodes the remaining subunits. mtDNA is located close to the mitochondrial inner membrane’s electron transport chain (ETC) and has no histone protection, antioxidant mechanism, or effective repair system. Reactive oxygen species (ROS) can easily cause cellular damage when ROS levels exceed the scavenging ability of the endogenous antioxidant defence system; oxidative stress results when there is oxidative/antioxidant imbalance. mtDNA is easily affected by ROS, and mtDNA copy number may be increased or decreased under oxidative stress.

## 2. Mitochondrial DNA and oocyte aging

### 2.1. Mitochondrial DNA copy number and oocyte aging

Normally, the oocyte is ovulated and fertilized while in metaphase II. The optimal fertilization time of the oocyte varies among species: for mice, it is at 8-12 hours after ovulation; for rabbits, 6-8 hours; and for both rhesus monkeys and humans, 24 hours. If the oocyte is not fertilized during the best fertilization period, the unfertilized oocyte remains in the fallopian tube and gradually enters the process of degradation and apoptosis known as post-ovulatory oocyte aging (Miao et al., 2009). In pre-ovulatory oocyte aging, related to maternal age, the aging process has begun but has not reached the stage of degradation. Increasing maternal age may result in the gradual decline of oocyte quality and developmental potential. Advanced maternal age leads to oocyte aging before, rather than after, ovulation, which results in abnormal fertilization and embryo development. In the present review, we mainly focus on pre-ovulatory aging.

Control of the mtDNA copy number is crucial for normal cellular function (Clay Montier et al., 2009). mtDNA is constantly replicating as the oocyte matures, but is stopped and the number is stable after maturation (meiosis II eggs). MPV17, an inner mitochondrial membrane protein in mammals and yeast, plays a role in maintaining mtDNA and oxidative phosphorylation activity (Dallabona et al., 2010). Mature metaphase II oocytes can contain 100,000 mitochondria and 50,000–1,500,000 copies of the mitochondrial genome (Monnot et al., 2013). mtDNA number stability suggests that mtDNA replication is silenced during pre-implantation development from the mature metaphase II oocytes to the pre-implantation embryo. It resumes between days 5 and 6 in murine embryos and continues until the blastocyst stage (Aiken et al., 2008). mtDNA copy number in metaphase II oocytes is being investigated as a marker of developmental competence (Chiaratti et al., 2010).

Over recent decades, the proportion of primipara women over the age of 35 has risen in the USA.

It has been shown that age-related ovarian follicle loss is nonlinear and accelerates with age, especially after 38 years old (Faddy, 2000). Neuroendocrine factors, uterine factors, and oocyte quality contribute to maternal age-related decline of successful pregnancy, but the consistent live-birth rate of pregnancies from oocyte donation in aging women suggests that decline in oocyte quality is the major contributing factor responsible for infertility with aging. Studies have underlined the correlation between mtDNA content and fertility, showing that mtDNA levels in human oocytes are inversely associated with maternal age and ovarian reserve indicators (Muller-Hocker et al., 1996; Steuerwald et al., 2000; Duran et al., 2011).

In a study that quantified mtDNA levels of first polar bodies (PBs) biopsied from oocytes from 27 women ranging in age from 30 to 45, PBs from older women tended to contain lower mtDNA quantities than those from younger women (Konstantinidis et al., 2014). Other studies have also indicated that mtDNA copy number in human oocytes decreases with advancing maternal age (Chan et al., 2005). Much of the decline in oocyte competence seen with age can be attributed to increasing aneuploidy rates, but it is conceivable that mitochondrial abnormalities may also play a role (Barritt et al., 2000).

Interestingly, the PB study simultaneously analysed mtDNA amounts in single -blastomere and trophoectoderm samples from the same women, and found that the older women had higher mtDNA amounts at these stages, though the differences did not reach statistical significance. This is consistent with a research that measured the amount of mtDNA in blastocysts (Diez-Juan et al., 2015). The research demonstrated that chromosomally normal blastocysts that failed to implant contain higher mtDNA copy number than those which implanted, with a threshold level of mtDNA, above which implantation never occurred. In addition, a number of studies have suggested that mtDNA levels are significantly higher in aneuploid embryos compared with those in chromosomally normal embryos (Tan et al., 2014; Fragouli et al., 2015). 

From the stage of oocyte to embryo, mtDNA level from older women presents an opposite trend. An interpretation of these findings is that the early embryo facilitates the propagation of mutant mtDNA. Once fertilization has succeeded, mtDNA number decrease sharply during preimplantation development (Spikings et al., 2007). Consequently, increased mtDNA amount in euploid embryos has been associated with poor implantation potential and may indicate reduced metabolic fuel during oocyte maturation (Diez-Juan et al., 2015). Generally speaking, increased mtDNA may be linked to elevated metabolism and are associated with reduced viability, a possibility consistent with the ‘quiet embryo’ hypothesis (Fragouli et al., 2015).

### 2.2. Mitochondrial DNA deletion and oocyte aging

The age-related incidence of mtDNA defects is closely related to ovarian reserve (Kitagawa et al., 1993; Suganuma et al., 1993). Oocytes can be dormant for over 40 years, and during this time they are exposed to harmful endogenous factors such as ROS and free radicals, which cause their mtDNA to cluster and mutate. A 4,977-bp deletion detected in some oocytes derived from ARA women (Fragouli et al., 2015) is the most common mtDNA deletion (Tanaka et al., 1989), likely because there is homology between the nuclear ATP enzyme 8 and mitochondrial MT-ND5 genes (ACCTCCCTCACCT). This deletion represents the loss of a large segment of mitochondrial genes, including genes encoding ATP synthases 6 and 8, cytochrome oxidase subunit 3 (COIII), NADH, and four subunits of the pan–reductase (ND3, ND4, ND4L, and ND5). This deficiency may lead to reduce overall expression of the missing genes as well as the production of the fusion gene product. It is speculated that, despite mtDNA mutations in a human oocyte, the mitochondrial bottleneck effect can decrease the mutation burden in individual cells of the embryo. In a study examining genes involved in mitochondrial biogenesis or key mitochondrial functions – such as apoptosis and antioxidant activity - in women with diminished ovarian reserve (DOR) compared to those with normal ovarian reserve (NOR), peroxisome proliferator-activated receptorgamma coactivator 1- α (PPARGC1A) was found to be down-regulated in DOR cumulus cells (CCs), but not in the NOR samples (Boucret et al., 2015). PPARGC1A regulates mitochondrial biogenesis and respiration (Puigserver et al., 1998; Wu et al., 1999). It was also found that oocytes and cumulus cells of NOR patients contained higher levels of mtDNA than those from DOR patients. 

### 2.3. Cell-free DNA and oocyte aging

DNA fragmentation is a result of apoptotic or necrotic events, and the resulting cell-free DNA (cfDNA) can be easily detected in blood and in body fluids (Ralla et al., 2014). cfDNA can also be actively secreted by cells (Gahan et al., 2008). Upregulation of cfDNA levels is found in some cancers and certain severe diseases, including gynaecological and obstetrical disorders (Swarup et al., 2007; Schwarzenbach et al., 2011; Traver et al., 2014). cfDNA is already used as a non-invasive biomarker for early detection and/or prognosis in these diseases.

Recent studies have evaluated cfDNA content in ovarian follicles and embryo culture medium (Stigliani et al., 2013; Scalici et al., 2014; Traver et al., 2015). In one, cfDNA fragments were found to be lower in follicular fluid (FF) samples from high-quality embryos compared to poor-quality embryos (Scalici et al., 2014). As well, markedly lower cfDNA levels were found in FF samples with embryos with low fragmentation rate (≤25%) compared to high fragmentation rate. cfDNA level in human follicular environments was also found to be significantly higher in FF samples from patients with ovarian reserve disorders. FF cfDNA level may thus be an independent and significant predictive factor for pregnancy outcome.

A biomarker of follicular microenvironment quality may be used to predict IVF prognosis and enhance female infertility management (Traver et al., 2015). The literature currently suggests that cellfree mtDNA (cf-mtDNA) is more meaningful than cfDNA, because it represents energy metabolism. One study found that, in 800 embryo culture medium samples analysed via real-time PCR, cf-mtDNA levels were significantly higher in fragmented embryo culture medium samples generated by women over 35 years than in those by younger women (Stigliani et al., 2013).If it is the case that cf-mtDNA levels in embryo culture medium are related to embryonic mtDNA levels, this could be used as a non-invasive pre-implantation genetic screening (PGS) method. Methods of detection of mtDNA levels in FF warrants further study, with its promise as a non-invasive approach to better assess pregnancy outcome.

## 3. Mitochondrial contributions to oocyte aging

Two PBs are released during oocyte formation. The process of moving chromosomes outside the oocyte to form the first and second PBs requires a significant amount of energy, provided by ATP from the mitochondria. As mitochondrial dysfunction is associated with oocyte aging (Bentov et al., 2011), this process can be expected to become impaired with age. Mammalian oocytes lack a robust spindle assembly checkpoint, which may explain in part the increased incidence of aneuploidy of maternal origin (Howe et al., 2013); indeed, research suggests that age-related spindle and chromosome abnormalities could contribute to the higher prevalence of aneuploidy in older women (Capalbo et al., 2013). Maternal aging is known to trigger a series of molecular alterations that drive the defects in chromatid separation (Chiang et al., 2011) and chromosome decondensation, as well as spindle detachment causing chromosomal misalignment (Battaglia et al., 1996; Liu et al., 2002). These molecular alterations are likely to be consequences of ATP deficiency.

### 3.1. CoQ and oocyte aging

The molecular changes in oocytes induced by aging are related to a loss of energy, which is normally provided by mitochondria and mostly via oxidative phosphorylation (OXPHOS) (Dumollard et al., 2007), as glycolysis, the alternative energetic process, is limited in the oocyte due to low expression of phosphofructokinase. Interference with OXPHOS or mitochondrial function impedes oocyte maturation, chromosomal misalignment and embryo development (Takeuchi et al., 2005; Thouas et al., 2006; Wyman et al., 2008). ATP production by OXPHOS involves the action of the electron transport chain, located on the inner mitochondrial membrane. Complexes I and II oxidize the tricarboxylic acid (TCA) cycle products and transfer the electrons to ubiquinone, also known as coenzyme Q (CoQ). The electrons are transferred to complexes III and then IV, producing a proton gradient which allows for ATP generation by complex V. CoQ has critical antioxidant properties, controls cellular redox, effects changes in various signalling pathways, and influences transcriptional activity (Crane, 2001) vital to the electron transport chain. Any decrease in the endogenous synthesis of CoQ results in respiratory chain dysfunction, and thus induces changes in membrane structure and dynamics that alter the environment in which mitochondrial enzymes and redox carrier molecules function. Upon condensation of the ring of CoQ with the polyprenyl tail by the CoQ2 enzyme, the ring structure is modified by decarboxylation, hydroxylation, and methylation, mediated by enzymes CoQ 3, 6, and 7. CoQ proteins form a large mitochondrial complex (Tran et al., 2007), and the presence of all protein components is required for stability (Wang et al., 2009). It has been shown that coenzyme Q10 supplementation in aged animal models can be used to delay the depletion of ovarian reserve, restore the expression of mitochondrial genes in oocytes, and improve the mitochondrial activity, but supplementation had no effect on ovarian reserve or quality in young animal models. It is therefore proposed that defects in mitochondrial performance accompany the decline in breeding performance, and that insufficient production of CoQ by oocytes plays an important role (Ben-Meir et al., 2015).

### 3.2. Mitochondrial membrane potential and oocyte aging

Many mitochondrial functions depend on the maintenance of membrane potential, including protein import, ATP generation, and lipid biogenesis (Bauer, 2014). In the normal quiescent condition, Na+ /K+ and Na+ /Ca2+ proton pumps create and stabilize membrane potentials. The proton pumps embedded in the mitochondrial inner membrane create a gradient by pumping protons from the mitochondrial matrix into the intermembrane space. The gradient is maintained by the relatively impermeable inner membrane. Generation of ATP depends on membrane potential stability, and ATP/ K+ channels in the mitochondrial membrane are implicated this stability. Pathological stimulation of cells may induce voltage-gated K+ channels opening, which leads to a change in mitochondrial membrane potential and an increase in mitochondrial Ca2+. Elevated Ca2+ can induce increased ROS and nitric oxide (NO) production, and also induces calciumdependent gene expression and the calciumdependent protein kinase activation that results in tumour cell proliferation (Repke, 1988).

Mitochondrial membrane potential determines many mitochondrial activities, and the spatial distribution of high-polarized and low-polarized mitochondria may reflect differential regulatory roles that, for the oocyte and early blastomere, may be focal and spatially distinct, but for the blastocyst may be cell-type specific. High-polarized mitochondria have been found to cluster, and they are able to maintain structural stability during oocyte maturation, fertilization, and the initial cleavage divisions (Van Blerkom et al., 2006). Wilding et al., measuring changes in the mitochondrial inner membrane potential of 2- to 3-day-old human embryos (Wilding et al., 2003), found a significant correlation between low membrane potential and a state of chaotic mosaicism, in which there was random segregation of chromosomes between the blastomeres. The chaotic mosaic embryos exhibited a slower cleavage rate and were significantly more common in the older patients.

### 3.3. Mitochondria number and oocyte aging

In mammalian cells, the number of mitochondria can vary significantly, from hundreds to many thousands, and is determined by the volume of the cell and its energy requirements. Granulosa cells from ARA women have been found to have a diminished number of mitochondria compared to their younger counterparts (Tatone et al., 2006). While too few mitochondria impede ATP generation, too many also affects cellular metabolism (Ylikallio et al., 2010). In mammals, mitochondria and mtDNA are inherited through the female germ line. Increasing ATP content is correlated with the oocyte maturity, and so decreased mitochondrial quantity as thought to be a factor that could explain the decreased oocyte. Duran et al. suggested that the ATP content of oocytes increases linearly until the final maturational stage at the time of lysis, excluding degenerated oocytes; however, no significant difference is seen in the number of mitochondria among the oocytes of different maturation stages (Duran et al., 2011). A stepwise multiple regression analysis to identify determinants of both ATP content and number of mitochondria, controlling for parameters that could simultaneously affect these measures, identified only final maturation stage as a predictor of ATP content. Old mitochondria are morphologically different and functionally inferior, producing more oxidants and less ATP, which may cause critical failures in energy-demanding cellular processes. This is confirmed by Zeng et al. who found that, as human oocytes proceeded through meiosis, ATP content increased (Zeng et al., 2007). Duran et al also found that mitochondrial numbers, predicted using mtDNA quantification, were more closely associated with FSH-predicted reproductive age than with chronological age (Duran et al., 2011). ATP content and FSH levels showed no statistically-significant correlation, but a moderate inverse correlation between FSH level and mtDNA number was observed, where the oocytes with the highest number of mtDNA copies were retrieved from patients with FSH levels 2.5-times lower than those with the lowest mtDNA numbers.

Some diseases affect oocyte mitochondria number and ATP content. A study exploring the effects of endometriosis on the follicular environment found that CCs of women with endometriosis had significantly less ATP production than the control group (Hsu et al., 2015). However, there were no obvious changes in mtDNA quantities between the groups. Endometriosis may thus cause CC mitochondrial dysfunction and lead to defective apoptosis and increased oxidative stress, which could in turn prevent the CCs from adequately supporting the developing oocyte they surround, affecting its function and the fertility of the patient.

### 3.4. Mitochondrial morphology and oocyte maturity

During the process of mammalian oocyte maturation, the mitochondria in the cytoplasm undergo spatial redistribution. Stojkovic et al. (2001) found a significant difference in mitochondrial distribution between immature and mature bovine oocytes: before in vitro maturation (IVM), mitochondria clusters were small and mitochondria activity was low, with the mitochondria distributed in the periphery of the cytoplasm; after IVM, the mitochondrial clusters became larger and the staining became deeper, with the mitochondria distributed centrally in the cytoplasm. Tarazona et al. (2006) confirmed these results. Nishi et al. using mouse oocytes, suggested that perinuclear mitochondrial aggregation is a component of cytoplasmic maturation, and a lack of this localization can lead to blockage of the maturation of mouse oocytes (Nishi et al., 2003). Brevini et al. (2005) also found that mitochondria in the most immature oocyte cytoplasm were peripherally distributed, and that in in vitro maturation, mitochondria in high-potential oocyte cytoplasms were evenly distributed, but were not redistributed in the low-potential oocytes. Hales et al. showed that active mitochondria in porcine oocytes at the germinal vesicle (GV) phase cluster in the cytoplasmic periphery, gradually shift to the central cytoplasm after 16 hours post-culture, and then localize to the perinuclear region after germinal vesicle breakdown (GVBD) (Hales, 2004). This same distribution change – from peripheral in the immature oocyte to clustered in the mature oocyte – has been noted in human oocytes (Liu et al., 2010). Interestingly, mitochondrial distribution has been found to slightly differ between in vivoand in vitro-matured oocytes: mitochondria of in vivo-matured oocytes were more abundant in the inner cytoplasm than in the peripheral region. Mitochondria distribution in IVM oocytes were more aggregated in periphery than in central of cytoplasm. In IVM oocytes, lack of mitochondrial distribution in the central region of the cytoplasm may be due to inadequate culture conditions. Mitochondria thus seem to use stage-appropriate distribution to participate in cell metabolism and apoptosis regulation.

Mitochondria are classically spherical in shape, with sparse cristae (Sathananthan et al., 2000), but their shape varies widely between cell types. In many adherent cell types, such as fibroblasts, mitochondria form a dynamic interconnected network, where short and long tubular mitochondria continuously divide and fuse (Chen et al., 2004). Several reports have demonstrated that the subcellular distribution of mitochondria during meiosis is mediated by microtubules (Van Blerkom, 1991; Yu et al., 2010; Dalton et al., 2013), but little attention has been given to the involvement of intrinsic mitochondrial dynamics in this process.

A mitochondrion’s function and efficiency are linked to the organelle’s morphology, which changes between organisms, tissues, and under different environmental conditions. Mitochondria usually take on a highly dynamic tubular-like or filamentous shape. Some studies have provided molecular insight into the mechanisms underlying mitochondrial fission and fusion, which are mediated by dynamin-like GTPases that are well-conserved among yeast, flies, and mammals (Okamoto et al., 2005). In mammals, fusion between mitochondrial outer membranes is mediated by mitofusin (Mfn) 1 and 2 (Santel et al., 2001; Chen et al., 2003; Santel et al., 2003), while fusion between the mitochondrial inner membranes is mediated by optic atrophy 1 (Opa1) (Olichon et al., 2003). Mitochondrial fission is mediated by dynamin-related protein-1 (Drp1), which is distributed in the cytoplasm and is recruited to the mitochondrial surface (Smirnova et al., 1998; Pitts et al., 1999). Mitochondrial fusion and fission are normally balanced processes, and overexpression of mitochondrial fusion or fission proteins affects mitochondrial morphology (Wakai et al., 2014).

MARCH5, a key protein controlling mitochondrial fission and fusion, was identified as a mitochondrial ubiquitin ligase that facilitates mitochondrial elongation (Park et al., 2010). In MARCH5 knockdown cells, a high degree of mitochondrial interconnection could be attained by both a lack of Drp1, which reduces fission activity, and increased Mfn1 levels, which enhances fusion activity. Elongated mitochondria induced by Mfn1 overexpression often form perinuclear aggregates in MARCH5-depleted cells, indicating that MARCH5 depletion facilitates the formation of elongated mitochondria, which cause cellular stress and forces the cell into senescence.

## 4. Improving ovarian quality

### 4.1. Culture medium composition

Numerous studies have analysed porcine follicular fluid (pFF) supplementation in maturation medium. The majority of studies have found pFF to be beneficial in in vitro embryo production systems (Funahashi et al., 1993; Daen et al., 1994; Vatzias et al., 1999). However, Mao et al. showed that the effect of pFF supplementation in IVM medium restrains mtDNA replication and oocyte meiotic maturation (Mao et al., 2012). Follicular fluid is a complex mixture of iron, steroid, peptide hormones, growth factors, and lipids that are locally produced and derived from the serum. The positive, negative, and negligible effects of supplementation of IVM medium with pFF seen by different groups may be caused by different properties of follicular fluid due to follicular size, physiological stage of follicles, and whether the donors were gilts or sows. Mao also showed that adding growth factors can enhance oocyte maturation compared with control (Mao et al., 2012). NRG1 supplementation was found to provoke mitochondrial replication, increased mtDNA copy number in meiosis II oocytes compared to GV oocytes, and increased blastocyst percentage in both parthenogenetic and IVF embryos. mtDNA copy number is likely an important consideration as we aim to improve oocyte quality and developmental competence in in vitro technologies.

Loss of MARCH5 mitochondrial E3 ubiquitin ligase induces cellular senescence through dynamin-related protein 1 and mitofusin 1.Silva et al exploring mitochondrial biogenesis stimulation of oocytes during IVM, found that the addition of the antioxidants alpha-lipoic acid (ALA; 10 mM), alpha-tocopherol (250 mM), hypotaurine (1 mM) and N-acetylcysteine (NAC; 1 mM), and sirtuin (100 ngmL1) to AntiOX embryo culture medium led to higher mitochondrial membrane potential and ATP levels and increased rate of development of blastocysts compared to the AntiOx without antioxidant supplementation after IVM and oocyte fertilization in aged (13.5 months) B6D2F1 female mice (Silva et al., 2015). Expression of genes associated with oxidative stress (PI3K, FOXO3A and GLRX2) was reduced in the antioxidant supplementation group. When embryos from young (6-8 weeks) CF1 females were cultured with the addition of only NAC and ALA, more blastocysts developed compared to the AntiOX-alone group. Antioxidant supplementation increased gene expression and embryo development of older female mice, whereas a down-regulated level of antioxidants during culture was beneficial to embryos from young mice. Sato et al. (2014) additionally identified that the addition of resveratrol to maturation medium up-regulates mitochondria biosynthesis in porcine oocytes, resveratrol increased the expression of SIRT1, supporting the hypothesis that decreased SIRT1 is a factor contributing to in vitro oocyte aging. However, resveratrol supplementation of maturation medium did not affect the mtDNA copy number in the oocytes. Supplementation with 10 µM of the proteasome inhibitor MG132, however, dramatically increased the amount of ubiquitinated proteins and the mtDNA copy number, by 12 and 14%, respectively. When resveratrol was added to the MG132-supplemented medium, the mtDNA copy number increased significantly. This effect was diminished by the addition of the SIRT1 inhibitor EX527. As has been previously described, the expression of SIRT1 is associated with oocyte mitochondria number, measured via mtDNA copy number. Activation of SIRT1 by resveratrol heightened the biosynthesis and degradation of mitochondria in oocytes, Accordingly, replenishing and improving mitochondrial function and the developmental ability of oocytes. The general effectiveness of supplement in media is yet to be proven, and further data is required for clinical use.

### 4.2. Cytoplasmic transfer

Numerous studies have shown that oocyte cytoplasmic transfer can alter the quality of oocytes after IVF-embryo transfer (ET) failure, improve the developmental potential of the embryo, and increase pregnancy rates (Dale et al., 2001; Zhang et al., 2015). Three approaches – pronuclear transfer, spindle chromosome complex transfer, and PB transfer – have been established, employing different methods of transferring genetic material. Pronuclear transfer is the removal of the pronucleus of the male and female after fertilization and its transfer to a donor zygote, which prevents mtDNA mutations from being propagated (Craven et al., 2010). Zhang (2016) reported the first live birth after spindle chromosomal complex transfer. Both of these methods have been shown reduce mutated mtDNA transmission from oocytes to pre-implantation embryos. A polar body contains few mitochondria and shares the same genomic material as an oocyte-PB transfer uses the first and second PBs, PB1 and PB2, to replace the genome of recipient eggs as an efficient approach of mitochondrial transfer (Wang et al., 2014). A minimal carryover of donor (patient) mtDNA genotype is expected in the reconstituted embryos and offspring produced by PB transfer.

Important in the success of oocyte cytoplasmic transfer is the transplantation of mitochondria with intact function. However, an ethical consideration arises during this process, as genetic material is likely to be transferred as well. The use of autologous mitochondria, i.e. mitochondria from the same patient, has been proposed as an answer to the ethical opposition associated with the use of genetically-distinct mitochondria from an unrelated donor and avoids theoretical clinical and biological issues potentially associated with heteroplasmy. Mitochondria can be removed from one group of a patient’s oocytes and transferred to another group (St John, 2014), or somatic mitochondria, such as from cumulus cells, could be transferred. Chappel et al. have even proposed the injection of mitochondria retrieved from oocyte “precursor” cells (Chappel, 2013). At the beginning of 2015, Great Britain took the lead in legalizing the transplantation of mitochondria (Wise, 2014). This intriguing strategy is just beginning to find clinical use, and it has been a promising one for patients with aged and poorquality oocytes.

## Conclusion

As the most important organelles in cytoplasm, mitochondria play a crucial role during the process of oocyte maturation and embryonic development. Mitochondrial dysfunction and mtDNA mutation affects normal oocyte development. Decreased number of mitochondria contributes to oocyte aging. Revealing the mechanism of oocyte maturation and aging by studying the role of mitochondria will help to welcome a new age in reproductive technology.
